# Lifestyle habits and impact of the Mediterranean diet on facial acne severity in French women: a case-control study

**DOI:** 10.1097/JW9.0000000000000017

**Published:** 2022-05-13

**Authors:** Laurie Ah-Thiane, Jean Michel Nguyen, Amir Khammari, Brigitte Dréno

**Affiliations:** a Service de Dermatologie, Université de Nantes, CHU Nantes, CRCINA, Nantes, France; b Service de Biostatistiques, PIMES Hôpital St Jacques, CHU Nantes, Nantes, France; c Laboratoire Immunodermatologie, Université Nantes, CHU Nantes, CRCINA, Nantes, France

**Keywords:** acne, GEA score, Mediterranean diet, PREDIMED

## Abstract

Acne is a common benign inflammatory disease, but it has a significant psychosocial impact. The role of the diet in the development of acne is controversial. Some daily foods such as milk and fast-release sugars tend to promote acne. The Mediterranean Diet (MD) is based on virgin olive oil and nuts that are rich in polyphenols with anti-inflammatory properties. The aim of this study was to assess an association between the adherence to the MD and the severity of facial acne in French women. A case-control observational study was conducted in Nantes Hospital (France). Based on a validated PREvención con DIeta MEDiterránean questionnaire, the adherence to the MD was assessed. The Global Evaluation Acne severity score was assessed by a trained dermatologist. Forty women with mild-to-severe acne and 40 control subjects were included. A global linear model identified a significant negative correlation between the severity of acne and the adherence to the MD in acne patients (regression coefficient = −0.17; *P* = 0.017). This was the first study conducted in France to investigate the relationship between the adherence to the MD and the severity of facial acne in women. This study confirmed the importance of using a holistic approach for acne management. Further studies are needed to confirm our findings.

**What is known about this subject with regards to women and their families?**

- Facial acne could lead to social eviction since the visual appearance has a major impact on social life.
- Women are more likely to take care of their skin through the use of cosmetics or specific diets.
- Some daily foods such as milk and fast-release sugars could contribute to the development of acne lesions.

**What is new from this article as messages for women and their families?**

- Adoption of a Mediterranean diet could be beneficial to women with moderate-to-severe acne.
- Contrary to preconceived ideas and based on our results, acne patients should be advised to reduce their wine consumption.
- A good adherence to a Mediterranean diet in women could also reflect a good adherence to medical treatment, resulting in better acne management.
- A higher socioeconomic status tended to be associated with less severe acne, suggesting that this could be a possible confounding factor affecting our results.

## Introduction

Acne is a very common and invalidating inflammatory disease of the pilosebaceous follicle. The inflammatory process underlying acne pathogenesis involves the innate immunity, in particular pattern recognition receptors such as the toll-like receptors, antimicrobial peptides, inflammatory cytokines such as interleukin (IL)-1, IL-6, tumor necrosis factor-α, and metalloproteases.^1^ Numerous local and systemic acne treatments are available and may be prescribed by physicians. However, the new generations of patients, especially women, are willing to be involved in their own medical care. Acne is a popular topic, taken up by the media, press reviews and also by independent writers. In bookshops, many authors encourage acne subjects to take care of their skin through the use of cosmetics, personal care products, and also specific diets.^2^ Thus, for dermatologists, the management of acne is not only to prescribe drugs but also to identify factors modulating acne lesions and, therefore, to promote a holistic approach for acne. The role of daily foods in acne development is controversial. Only one study assessing acne and diets has been conducted in France: as part of this individual survey, the Conseil Supérieur de l’Audiovisuel Santé has developed a food questionnaire to be completed by the French population and an association between chocolate and sugar consumption, and acne development has been highlighted.^3^ However, there was a methodological bias because acne severity was self-assessed by the patients and only affected patients who participated in the survey.

Besides, in France, the adherence to the Mediterranean Diet (MD) has been studied more generally using a validated 14-item questionnaire from the PREDIMED (PREvención con DIeta MEDiterránean, http://links.lww.com/IJWD/A7) study.^4^ This questionnaire was initially used in patients with cardiovascular disorders and the MD has been shown to reduce the incidence of these diseases through its anti-inflammatory properties.^5^ This particular diet promotes the consumption of fruits, vegetables, whole grains, nuts, and virgin olive oil.^6^ The MD also includes a moderate consumption of wine, fish, white meat, and a restricted consumption of red meat and sugary products.

To date, no study has investigated the effects of the MD in French women with facial acne. The aim of this study was to determine whether there is a relationship between facial acne severity and the adherence to the MD in France.

## Materials and methods

A case-control observational study was conducted in the dermatology department of Nantes Hospital. The study was not supported by the pharmaceutical industry. All patients provided a signed informed consent and the study was approved by our local ethics committee (Groupe Nantais d’Ethique dans le Domaine de la Santé).

Forty female patients with different severities of acne were consecutively included from a pool of patients referred for the first time to our dermatology department between July 1 and December 31, 2020. Exclusion criteria were patients with only truncal acne and without facial lesions because the Global Evaluation Acne (GEA) scale^7^ only rates facial acne, patients under guardianship or curatorship, patients with corticosteroid-induced acne and patients previously treated with isotretinoin. Forty age- and body mass index (BMI)-matched healthy subjects from the same geographic area (near Nantes, France) were selected as controls among patients referred to our dermatology outpatient department for another reason (except for hidradenitis suppurativa [HS] since a recent paper has shown a continuum between HS and conglobata facial acne^8^). Data collected included the age, weight, height, BMI, consumption of fast-release sugars and dairy products, snacking habits, family history of acne, smoking status, alcohol consumption except wine (because wine consumption is traditionally advised in the MD^9^), the use of cosmetics and a previous use (≥1 month before the study visit) of local and systemic treatments. The socioeconomic status of all patients was also determined using the modified Kuppuswamy scale.^10^ In patients who initiated antiacne treatment ≥1 month before the study visit, the adherence to medical treatment was assessed using the ECOB questionnaire developed by Pawin et al.^11^ that has an 89% specificity for detecting a poor adherence.

A validated 14-item PREDIMED^4^ questionnaire, http://links.lww.com/IJWD/A7, was used to assess the adherence to the MD in all patients and subjects during the consultation. For each item, a score of 1 or 0 was assigned. A total PREDIMED score ranging between 0 and 5 corresponded to a low adherence; between 6 and 9 to an intermediate adherence; and ≥10 to a high adherence. For each patient with acne, acne severity was evaluated by a dermatologist who was blinded to the study design to prevent rating biases. The GEA scale was used to measure the clinical severity of acne.^7^

### Statistical analysis

Results are presented as a mean ± standard deviation. Data distribution was assessed using a bivariate analysis to compare the acne group and the control group. Differences in several parameters in acne patients were analyzed using a Spearman correlation and Wilcoxon rank tests. A General Linear Model was used to take into account other potential confounding factors. An α level less than 0.05 was considered significant. Data were recorded using Excel Microsoft (Version 2010) and analyzed using R Software (Version 3.8).

## Results

A total of 80 women were included, 40 acne patients and 40 control subjects. In acne patients, the mean GEA score was 2.85 ± 0.7, the mean PREDIMED score was 5.7 ± 1.8, the mean age was 19.75 ± 4.3 years, and the mean BMI was 21.8 ± 3.1 kg/m^2^.

The comparison between acne patients and control subjects confirmed a good matching for the age and BMI. A significant difference in family history of acne was found (*P* = 0.0006; odds ratio = 5.4; 95% confidence interval = [1.92–16.5]).

### Correlation between the GEA score and the PREDIMED score

**Fig. 1.**
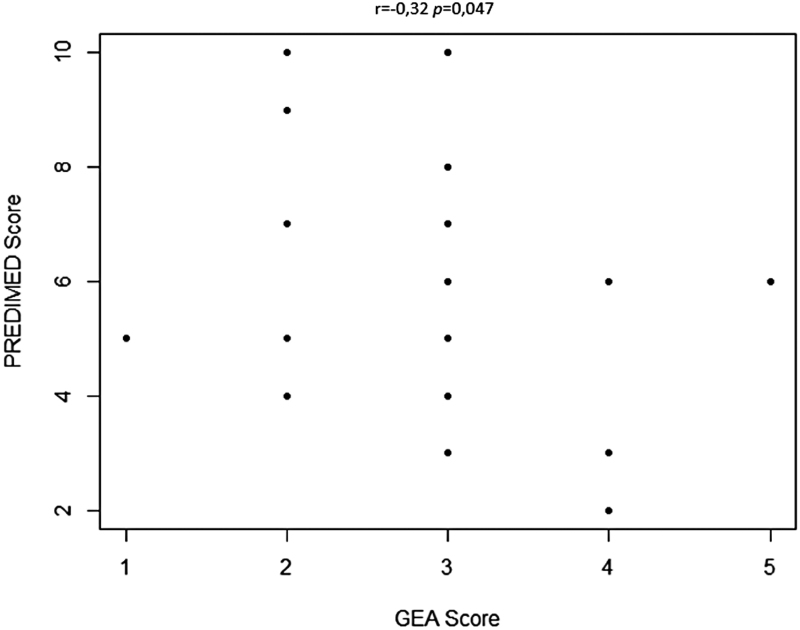
Correlation between the GEA score and the PREDIMED score in acne patients. A significant negative correlation was found between the severity of acne determined using the GEA score and the adherence to the Mediterranean diet (*P* = 0.047, Spearman’s rank correlation).

In acne patients, a significant negative correlation was found between the severity of acne determined using the GEA score and the adherence to the MD (*P* = 0.047, Spearman’s rank correlation) (Fig. 1). This negative correlation was confirmed in an analysis including all acne patients and control subjects (Spearman correlation coefficient r = −0.47; *P* <0.001).

### Correlations between the lifestyle habits and the PREDIMED score

No significant association was found between the adherence to the MD and the BMI and the studied qualitative factors (consumption of fast-release sugars and dairy products, snacking habits, family history of acne, smoking status, alcohol consumption, use of cosmetics, and previous use of local and systemic treatments). Only the age significantly and positively correlated with the adherence to the MD.

In acne patients, the General Linear Model adjusted for other factors showed a significant decrease in GEA score with the decrease in PREDIMED score (multiple regression coefficient = −0.17; *P*=0.017).

A subgroup analysis was performed using the results of question 8 from the PREDIMED questionnaire, http://links.lww.com/IJWD/A7 regarding wine consumption, and a significant positive correlation was found between the consumption of more than seven glasses of wine per week and the severity of acne (*P* = 0.012).

### Correlations between the lifestyle habits and the GEA score

No significant association was found between the severity of acne and the BMI and the studied qualitative factors (consumption of fast-release sugars and dairy products, snaking habits, family history of acne, smoking status, alcohol consumption, use of cosmetics, and previous use of local and systemic treatments).

### Correlation between the adherence to the MD and the adherence to medical treatment

A subgroup analysis was performed in the 33 women who received a topical or oral treatment. A correlation was found between a good adherence to the MD and a good adherence to medical treatment, without reaching significance (*P* = 0.055).

### Correlation between the adherence to the MD and the socioeconomic status

In the whole cohort, nonsignificant association was found between the socioeconomic status and the adherence to the MD (*P* = 0.07).

### Factors limiting the adoption of the MD

At the end of the PREDIMED questionnaire, http://links.lww.com/IJWD/A7, patients were asked what could explain a low adherence to such a diet. Twenty-eight patients (35%) reported not being able to follow this diet due to its cost, 24 patients (30%) reported that the lack of time to buy food and cook could be an obstacle to follow a specific diet, and 43 patients (30 minors and 13 adults) did not know what this diet was about. Eleven parents of minor patients reported having difficulties to answer some questions because half of the meals of their child were eaten at school and 24 minor patients (75% of them) reported that their diet was chosen by their parents.

## Discussion

In this study, we showed that acne women with a lower adherence to the MD experienced more severe acne. Therefore, adopting a specific diet such as the MD could help to reduce acne severity in acne patients, as previously suggested in an Italian study.^12^

In the MD, wine consumption is recommended because of its phenolic composition. However, we interestingly found more severe cases of acne in patients consuming more than seven glasses of wine per week. Therefore, contrary to preconceived ideas and based on our results, acne patients should be advised to reduce their wine consumption. However, we did not collect the exact number of glasses consumed per week, so what we could not precisely assess the dose–effect relationship with wine consumption.

Another additional point highlighted in our study was the importance of the overall adherence to all proposed acne management strategies combining lifestyle changes, dietary rules, and medical treatment. We showed an association between a good adherence to the MD and a good adherence to treatment. Patients with a poor adherence to medical treatment tended to be less adherent to the MD. It could be interesting to explore this finding in other studies aiming to assess the overall difficulty in observing and adhering to acne management.

In our study, we identified potential barriers to follow the MD, including the cost, the required time, the unawareness of this diet and the difficulty to monitor such a diet, in particular in younger patients (who eat half of their meals at school or who eat what their parents chose for them). Regarding the cost, the adherence to the MD tended to be higher in our patients with a higher socioeconomic status, although our results were not significant. Thus, the socioeconomic status could be a potential confounding factor for the adherence to the MD. However, the socioeconomic status was difficult to assess and the ECOB questionnaire was based on patient-reported outcomes, but this is an easy method that can be used in routine daily practice.

In addition, the BMI could be one of the factors influencing acne severity. However, we did not find any significant association, in line with Gayen et al.^13^ who have studied the association between the metabolic status and obesity and acne severity. Our overall small sample size could explain why none of the other studied factors, especially dairy product and fast-release sugar consumption, significantly influenced acne severity. This finding is in contrast with recent studies that have shown a significant association between acne and high glycemic load diets.^14^ Indeed, high sugar levels have been shown to increase insulin and IGF1 release, leading to androgen secretion, sebum overproduction, and keratinocyte proliferation.^15,16^ The hormones found in milk can also increase IGF1 levels.^17^ In our study, 46% of participants reported a daily consumption of dairy products equivalent to one glass of milk, which is low compared to other studies. Indeed, in a Norway study, 72% of the population consumed semiskimmed dairy products.^18^ Finally, heredity is considered a predictive factor for acne risk and severity,^19^ as confirmed in our study with a significant difference in family history between our acne patients and our control subjects (*P* = 0.0006). We showed that having at least one parent with a history of acne increased by 5.4 the risk of developing acne compared to nonacneic patients. Dréno et al.^20^ have shown that a family history of acne was a predictive factor for acne severity.

Barrea et al.^21^ have performed a similar analysis in Naples, but they have focused on HS instead of acne. In their study conducted in 41 patients, they have shown a relationship between the severity of HS and the adherence to the MD, and a poor adherence to the MD correlated with grade 2 or 3 HS,^21^ regardless of the gender, age, and BMI. The main interest of the MD is that it is not focused on a single nutrient but includes a whole alimentary group comprising virgin olive oil, fruits, vegetables, and red wine, which are rich in monounsaturated fatty acids and polyphenols, known for their antioxidant and anti-inflammatory properties.^22^

The main limitations of our study were its small sample size and its monocentric design. Indeed, Nantes is not located in the Mediterranean area which could explain the small number of subjects who strongly adhered to the MD. The vegetarian diet was not taken into account, and this could have impacted some answers. Also, we did not perform any biological analysis to identify inflammatory markers. Finally, our study was only based on a single consultation, but it could be interesting to analyze the kinetics of inflammatory markers to further assess the anti-inflammatory properties of the MD.

This first study conducted in France based on the effect of the MD in women with facial acne confirmed the importance of using a holistic approach for acne management. In association with a good adherence to treatment, the MD could be suggested as a hygienic and dietary rule, in particular in patients with moderate or severe frequently relapsing acne. However, further studies are needed to confirm our preliminary findings.

**Table 1.** Differences in lifestyle habits between acne patients and control subjects

Parameters	Acne patients, *N* = 40	Control subjects, *N* = 40	*P*
Age (years)	19.75 ± 4.3	19.75 ± 4.3	1
BMI (kg/m^2^)	21.8 ± 3.1	21.4 ± 2.7	0.56
Smoking (yes)	7	8	1
Alcohol consumption (>7 units per week)	2	0	0.49
Family history (yes)	30	15	**0.0006 OR = 5.4 [1.92–16.5]**
Dairy products consumption (equivalent to ≥1 glass per day) (yes)	18	19	1
Fast-release sugar consumption (yes)	24	19	0.36
Snacking (yes)	8	16	0.09
Cosmetic skin care use (yes)	16	6	**0.02**
Prior systemic treatments (yes)			
*Local	*29	*0	**<0.001**
*Systemic	*23	*0	**<0.001**

**Table 2.** Correlation between the lifestyle habits and the PREDIMED score

	PREDIMED score	
	*P*	Correlation coefficient
Age, years	**0.03**	**0.34**
BMI	0.19	0.21
Smoking	0.72	—
Alcohol consumption (except wine)	0.41	—
Family history	0.81	—
Dairy products consumption	0.47	—
Sugar consumption	0.73	—
Snaking	0.73	—
Use of cosmetics	0.056	—
Prior local treatment	0.052	—
Prior systemic treatment	0.26	—

BMI, body mass index; PREDIMED, PREvención con DIeta MEDiterránean.

**Table 3.** Correlation between the lifestyle habits and the GEA score in acne patients

	GEA score	
	*P*	Correlation coefficient
Age, years	0.59	−0.08
BMI	0.47	−0.11
Smoking	0.08	—
Alcohol consumption (except wine)	0.49	—
Family history	0.65	—
Dairy products consumption	0.23	—
Sugar consumption	0.44	—
Snaking	0.20	—
Use of cosmetics	0.69	—
Prior local treatment	0.37	—
Prior systemic treatment	0.42	—

BMI, body mass index; GEA, Global Evaluation Acne.

## Conflicts of interest

None

## Funding

None.

## Supplementary Material


