# Supplementary material for: Lifestyle habits and impact of the Mediterranean diet on facial acne severity in French women: a case-control study
Source: Int J Womens Dermatol. 2022 May 13;8(2):e017. doi: 10.1097/JW9.0000000000000017 (PMC9249266; doi:10.1097/JW9.0000000000000017)
Supplement: Supplementary file 1 [file jw9-8-e017-s001.pdf]

Validated 14-item Questionnaire of Mediterranean diet adherence.

Questions	Criteria for 1 point
1. Do you use olive oil as main culinary fat?	Yes
2. How much olive oil do you consume in a given day (including oil used for frying, salads, out-of-house meals, etc.)?	≥4 tbsp
3. How many vegetable servings do you consume per day? (1 serving : 200 g [consider side dishes as half a serving])	≥2 (≥1 portion raw or as a salad)
4. How many fruit units (including natural fruit juices) do you consume per day?	≥3
5. How many servings of red meat, hamburger, or meat products (ham, sausage, etc.) do you consume per day? (1 serving: 100–150 g)	<1
6. How many servings of butter, margarine, or cream do you consume per day? (1 serving: 12 g)	<1
7. How many sweet or carbonated beverages do you drink per day?	<1
8. How much wine do you drink per week?	≥7 glasses
9. How many servings of legumes do you consume per week? (1 serving : 150 g)	≥3

Questions	Criteria for 1 point
10. How many servings of fish or shellfish do you consume per week? (1 serving 100–150 g of fish or 4–5 units or 200 g of shellfish)	≥3
11. How many times per week do you consume commercial sweets or pastries (not homemade), such as cakes, cookies, biscuits, orcustard?	<3
12. How many servings of nuts (including peanuts) do you consume per week? (1 serving 30 g)	≥3
13. Do you preferentially consume chicken, turkey, or rabbit meat instead of veal, pork, hamburger, or sausage?	Yes
14. How many times per week do you consume vegetables, pasta, rice, or other dishes seasoned with sofrito (sauce made with tomatoand onion, leek, or garlic and simmered with olive oil)?	≥2

Appendix: ECOB Questionnaire

Oral treatment	Topical treatment
Do you remember the name of the last drugs you took? Yes	Do you remember the name of the last drugs you took? Yes
Have you used these drugs? Yes	Have you tolerated these drugs well? Yes
Have you forgotten to take these drugs at any time during the treatment period? No	Have you ever stopped taking these drugs because you thought it would do more harm than good? No
Have these drugs improved your acne? Yes	Have these drugs been useful for you? Yes

- If at least *one* answer is different from the expected answers (written in red above), the patient's adherence is considered to be poor.
- The 'adherence scale' based on this selection can be considered as a help to measure the risk of poor adherence, but does not allow an exact determination of the adherence, which, by fact, is unknown.

List of tables

Parameters	Acne Patients N = 40	Control subjects N= 40	p-Value
Age (years)	19.75 ± 4.3	19.75 ± 4.3	1
BMI (kg/m^2)	21.8 ± 3.1	21.4 ± 2.7	0.56
Smoking (yes)	7	8	1
Alcohol consumption (>7 units per week)	2	0	0.49
Family history (yes)	30	15	**0.0006 OR=5.4[1.92-16.5]**
Dairy products consumption (equivalent to ≥1 glass per day) (yes)	18	19	1
Fast-release sugar consumption (yes)	24	19	0.36
Snacking (yes)	8	16	0.09
Cosmetic skin care use (yes)	16	6	**0.02**
Prior systemic treatments (yes)			
*local	*29	*0	**< 0.001**
*systemic	*23	*0	**< 0.001**

Table 1: Differences in lifestyle habits between acne patients and control subjects

	Predimed score	
	p-Value	Correlation coefficient
Age	**0.03**	**0.34**
BMI	0.19	0.21
Smoking	0.72	-
Alcohol consumption (except wine)	0.41	-
Family history	0.81	-
Dairy products consumption	0.47	-
Sugar consumption	0.73	-
Snaking	0.73	-
Use of cosmetics	0.056	-
Prior local treatment	0.052	-
Prior systemic treatment	0.26	-

Table 2: Correlation between the lifestyle habits and the PREDIMED score

	GEA score	
	p-Value	Correlation coefficient
Age	0.59	-0.08
BMI	0.47	-0.11
Smoking	0.08	-
Alcohol consumption (except wine)	0.49	-
Family history	0.65	-
Dairy products consumption	0.23	-
Sugar consumption	0.44	-
Snaking	0.20	-
Use of cosmetics	0.69	-
Prior local treatment	0.37	-
Prior systemic treatment	0.42	-

Table 3: Correlation between the lifestyle habits and the GEA score in acne patients
